# Response of infrared thermography related parameters to (non-)sport specific exercise and relationship with internal load parameters in individual and team sport athletes—a systematic review

**DOI:** 10.3389/fspor.2024.1479608

**Published:** 2024-12-13

**Authors:** Lukas Masur, Florian Brand, Peter Düking

**Affiliations:** ^1^ Department of Sports Science and Movement Pedagogy, Technische Universität Braunschweig, Braunschweig, Germany; ^2^ Clinic for Trauma Surgery and Orthopedics, Städtisches Klinikum Braunschweig, Braunschweig, Germany

**Keywords:** individualization, precision training, muscle, inflammation, signature

## Abstract

**Introduction:**

Monitoring internal load is crucial for athletes but often requires invasive methods for muscle-related parameters, limiting practicality. Infrared thermography (IRT) related parameters might overcome this limitation. This systematic review aimed to examine the available literature on the response of IRT related parameters to (non-)sport specific exercise and reveal relationships with internal load parameters in athletic populations.

**Methods:**

Four scientific databases were systematically searched (February 2024) with keywords related to IRT, load, and sports disciplines. Risk of bias was evaluated using QUADAS-2. Main inclusion criteria for studies were i) reporting of IRT related parameters and other internal load parameters prior/post (non-)sport specific exercise ii) inclusion of least Tier 2 athletes ≥ 18 years. After identifying *n* = 10,538 studies, 13 articles (*n* = 231 participants) were included.

**Results:**

Following (non-)sport specific exercise in athletic populations, the majority of relevant studies showed a decrease in IRT related parameters within 15 min, while studies showed an increase in IRT related parameters following 30 min, 24 h, 48 h, and 72 h after exercise cessation. Relationships between alterations in IRT related parameters and other internal load parameters are inconsistent across the literature.

**Conclusion:**

While the majority of studies show an increase in IRT related parameters following (non-)sport specific exercise, relationships with other internal load parameters and underlying physiological mechanisms evoking IRT related alterations are not conclusively revealed in athletic populations. Future research needs to assess the relationship of IRT related parameters especially with inflammatory parameters in athletic populations following (non-)sport specific exercise. Practitioners might assess IRT related parameters in conjunction with other load parameters.

## 1 Introduction

Quantifying internal load by monitoring of various parameters holds a key role to individualize training procedures to advert fatigue, mitigate the risk of illness and injury, and optimizing performance outcomes (1). In this context, internal load pertains to an individual's psychophysiological response to the external load, which can be defined as the executed mechanical work (1). Depending on the bodily system at question, different methodologies are available to practitioners to assess internal load of athletes (2–4). However, these methodologies are often intrusive and/or time-consuming, particularly when muscle related parameters should be evaluated (4, 5). This limits the assessment of muscle related parameters in practice. Consequently, other approaches are needed which overcome these limitations.

To assess muscle related parameters, non-invasive infrared thermography (IRT) following exercise (6) is increasingly used in athletic populations (7–10) as well as in the medical literature (8, 11). IRT is a non-radiating, contact-free, and non-invasive approach to measure skin temperature and derive different parameters (e.g., skin temperature asymmetries, changes in skin temperature) (12). While healthy subjects are anticipated to maintain thermal equilibrium under neutral conditions (13), the metabolic, biomechanical, and physiological demands associated with training and competitive activities may elicit fluctuations in skin temperature after exercise cessation (12). The underlying mechanisms of the change of body surface radiation during and after exercise relies on several muscular and physiological factors, including increased ATP-production, neuronal responses, vasomotor adjustments and inflammatory processes (14–18). The specifics of these mechanisms, including their dependence on exercise type, are eloquently explained elsewhere (14, 17, 18).

Given the link between IRT and physiological mechanisms, research explored whether changes in skin temperature are associated with physiological parameters. Studies indicated that IRT-related parameters correlate with performance metrics, such as maximal oxygen uptake and heart rate (19, 20). Information about skin temperature alterations following exercise has been used to detect skeletal muscle overload and fatigue in athletic populations (21). Additionally, it was shown that if IRT related parameters are used to identify players at potential risk, injuries could be reduced in elite soccer players (21, 22).

However, despite isolated studies, there is no systematic review available in the literature on the response of IRT related parameters in response to (non-)sport specific exercise. To provide a stronger evidence base to inform sports practice and future research, the aim of this article was to systematically review available literature on the response of IRT related parameters to (non-)sport specific exercise and relationship with internal load parameter.

## 2 Methods

### 2.1 Study design

To be considered for inclusion, IRT related parameters must have been calculated from infrared thermographic images and must have been captured either pre and post, or only post (non-)sport specific exercise or competition in compliance to the Glamorgan Protocol (2) and/or the contemporary consensus statement recommendations delineated by Thermographic Imaging in Sports and Exercise Medicine (TISEM) for the measurement of human skin temperature (23). Studies were excluded if they did not align with these scientific recommendations.

Studies that assessed IRT related parameters preceding or concurrently with exercise, which involved consciously or unconsciously manipulated experimental conditions (e.g., environmental temperature), or which assessed methodological differences while obtaining IRT related parameters were out of the scope of this article and therefore excluded. Investigations where training was coupled with a manipulating experimental intervention (e.g., use of ergogenic aids, assessment of recovery procedures) were excluded.

### 2.2 Study populations

All investigations involving adult, healthy, able-bodied trained/developmental (at least Tier 2) team sport or individual sport athletes (24), regardless of sex or gender were included. As age is considered an influencing factor on thermographic parameters (25), and it has been demonstrated that skin temperature stabilizes after puberty (26), this review focuses on investigations incorporating athletes with a mean age of ≥18 years. Research involving non-human participants or participants with a mean age below 18 years was excluded. Studies with injured team sport players which are e.g., in the return to sport or return to play procedures were excluded.

### 2.3 Outcomes

In order to be included, the study must have examined IRT related parameters following (non-) sport specific exercise and the relationship between IRT related parameters (e.g., skin temperature; surface radiation temperature; skin temperature asymmetries) and at least one other internal load parameter in either the time, frequency or concentration domain (e.g., heart rate, ratings of perceived exertion, lactate). Accordingly, studies that measured skin temperature but did not integrate IRT-related parameters and/or internal load parameters were excluded.

### 2.4 Publication status and language

Only full-length original articles published in English in peer-reviewed journals will be considered, omitting “grey” literature such as conference abstracts, dissertations, theses, or reports. In addition, the reference lists of articles initially included were examined for additional publications of potential relevance. Articles published in other languages were excluded.

### 2.5 Search strategy

In accordance with the Preferred Reporting Items for Systematic Reviews and Meta-Analyses (PRISMA) guidelines (27) and the PRISMA 2020 Checklist (Supplementary Table S1), we conducted a systematic search of Ovid MEDLINE In-Process and Other Non-Indexed Citations, CINAHL, EMBASE, and Web of Science (with no restrictions on publication date) in February 2024 to identify potentially relevant articles using the search criteria outlined in Supplementary Table S2. The development of search terms and medical subject headings (MeSH) was conducted in collaboration with a skilled librarian. In addition, reference lists of identified articles were screened to detect pertinent articles that might have been failed to notice using this search profile.

### 2.6 Selection of articles

After importing potentially relevant articles into Citavi 6 (QSR International, Burlington, MA, USA) and removing duplicates, one of the authors screened the titles and abstracts based on our inclusion criteria, while a second investigator independently validated those evaluations. Subsequently, both individuals thoroughly reviewed the full texts of the relevant articles to assess eligibility (with awareness of the journals and authors involved). Discrepancies were resolved through discussion between authors, until a consensus was reached.

### 2.7 Data extraction and analysis

As previously performed (28), the process of data extraction from each article identified was divided into the following steps: (1) study characteristics by the publication details (authors, journal, date), sports, participants (mean age, number, sex), level of performance; (2) study-defined training or competition characteristics, data and time point related to internal load parameters; (3) data and time points related to thermographic parameters, analyzed body region of the thermographic assessment; (4) methodological approaches of the thermographic analysis by extracting the camera type, methodological analysis method of thermographic parameters; (5) statistical analysis.

### 2.8 Data synthesis

To evaluate the magnitude of the effects as performed in previously published research (29), percentage changes (*Δ*%) were calculated and illustrated in Figure 2, Tables 3 and 4 for study outcomes using the following equation:

$$\Delta \text{\%}\;=\;\frac{(\mathrm{Mpost}\;-\;\mathrm{Mpre})\;}{\mathrm{Mpre}}\;\times 100$$

Mpost represents the mean value after (long-term) training or competition and Mpre the baseline mean value.

Depicted mean values were calculated to summarize results of studies using standard equation, as follows:

$$\mathrm{Mean}\;\mathrm{value}\;=\;\frac{\sum \mathrm{values}}{\mathrm{number}\;\mathrm{of}\;\mathrm{values}}$$

### 2.9 Assessment of methodological quality

As recommended by Whiting et al. (30) and employed in similar research (11, 31), two experienced raters independently assessed the methodological quality using the QUADAS-2 scale, which comprises two domains: risk of bias and applicability (30, 32). The risk of bias domain evaluates items such as “patient or sample selection”, “index test”, “reference standard”, and “flow and timing”. The applicability domain assesses parameters including “sample selection”, “index test”, and “reference standard”. Regarding phase two of QUADAS-2, we tailored our review by omitting signal questions pertaining to blinding, as recommended in the official background document for objective index tests (30). Informed by fundamental principles, research, and recommendations (30, 32), we incorporated an additional signal question concerning patient/sample selection: “Does the study delineate inclusion and exclusion criteria for the selection process?”. Appropriate criteria were derived from Fernandez-Cuevas et al., 2015 (25) and involve medical history (injury, diseases, operations), intake factors (drug treatment, medicaments, alcohol, tobacco, stimulants) and application factors (ointments, cosmetics, therapies). Disagreements between raters were resolved through consensus.

## 3 Results

### 3.1 Study characteristics

**Figure 1:**
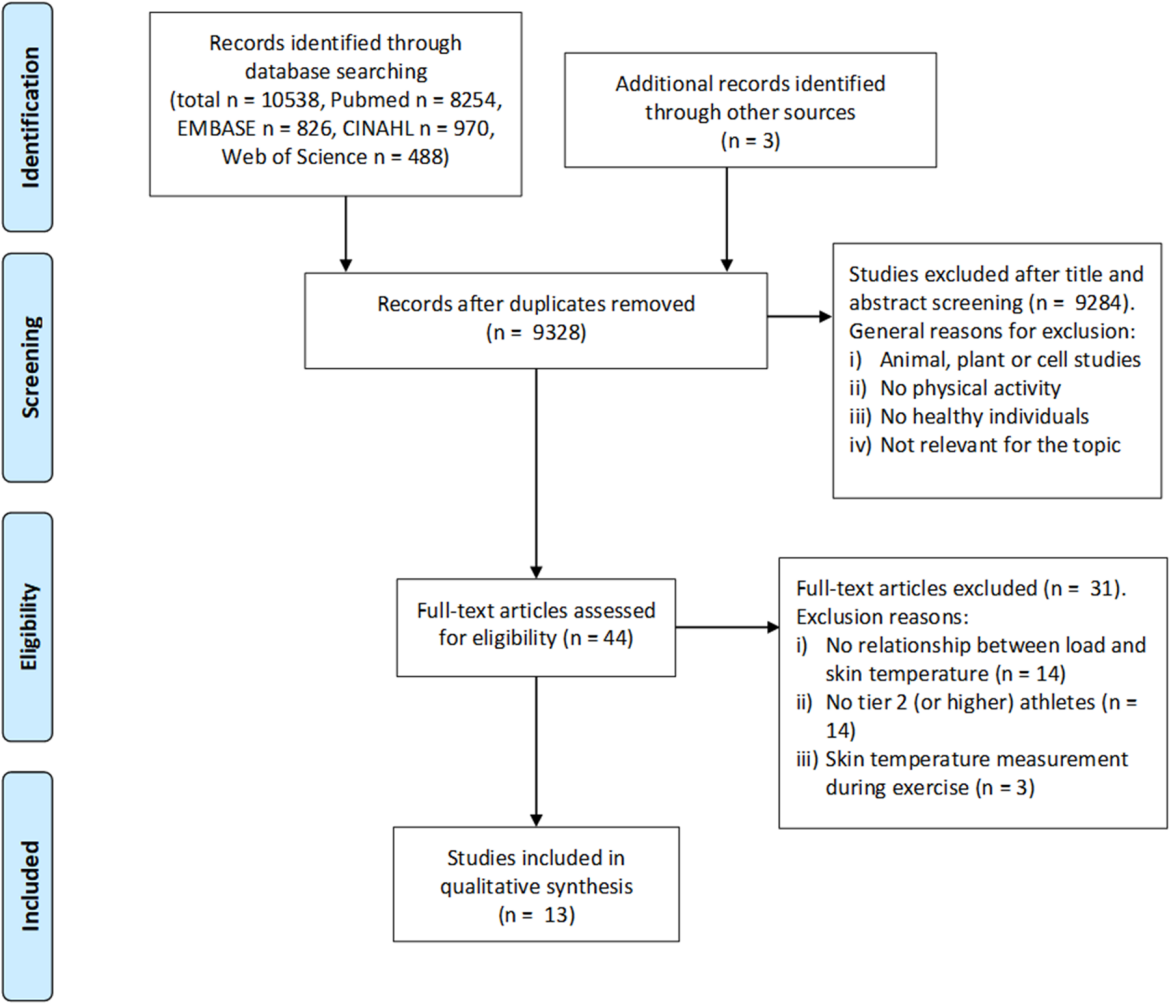
Process of study selection, from initial identification as potentially relevant to inclusion.

The compilation of records identified and examined is illustrated in Figure 1. A total of *n* = 10,538 studies were identified (PubMed *n* = 8,254; EMBASE *n* = 826, CINAHL *n* = 970, Web of Science *n* = 488). 13 articles conformed to our inclusion criteria, including 231 participants (193 males, 28 females; 10 non-disclosed participants; Age range: 18 to 41 years). Ten of 13 studies included *n* = 188 tier 3 athletes (3, 4, 7, 9, 12, 33–37) and three included *n* = 43 tier 2 athletes (38–40).

Eleven (7, 9, 12, 33–40) of 13 studies were published in between 2019 and 2024. Six studies were conducted in soccer (3, 4, 9, 12, 33, 36), three in endurance sports such as half-marathon (38), marathon (39), triathlon (40), two in judo (7, 37), one in sprinting (34) and one examined sprinters and endurance athletes (triathletes and long-distance runners) (35).

Tables 1, 2 summarize study characteristics including e.g., training or competition characteristics, load parameter description and methodological approaches.

**Table 1:** Characterization of the studies analyzed.

Sport	Article	N (male, female)	Age (years)	Level of performance	Study-defined training/competition characteristics	Load parameters	Time point of internal load measurement
Studies investigating response to a (partial) season; Soccer	(9)	22 (22,0)	27.7 ± 3.93	National C series championship	19 matches and 19 weeks with an interval of 7 days between matches	Blood creatine kinase concentration, athlete's perception of recovery, fatigue and pain	Post: 48 h
	(36)	20 (20,0)	Group 1: 25.6 ± 4.0; above total distance median Group 2: 27.7 ± 4.1; below total distance median	First Brazilian National Soccer League	Competitive season (16 weeks)	Blood concentration of creatine kinase, C-reactive protein, cortisol (no relationship/correlation calculated), rate of force development, impulse, peak force	Pre: Season Post: Season after an interval of 72-h of inactivity (no training)
	(12)	30 (30,0)	25.37 ± 3.60	Spanish second football division	12-week competitive period, training (five times/week) and match-play (one time/week)	Well-being questionnaire with following variables: (1) Modified version of Borg Rating of Perceived Exertion (RPE) (2) Stress (3) Rest time (4) Rest quality (5) Muscle Soreness	Pre: before training/competition (detailed time point n. r.)
Studies investigating response to a (partial) season; Other sports, Sprinting, Taper period	(34)	17 (9,8)	Male: 26.1 ± 3.3 Female: 25.1 ± 2.7	Elite; Polish National Team	10 d period consists of: Resistance training, speed-power training, speed-power training—relay runs, speed intervals, speed-power training (block starts), endurance training	Blood creatine kinase concentration	08:00 and 09:00 and between 20:00 and 21:00 local time; approximately Pre: 1 h, Post: not derivable
Soccer; Studies investigating response to ≤3 soccer games	(3)	1 (1,0)	27	First Division Brazilian Soccer League	Full official match	Blood/Plasma creatine kinase concentration	Pre: 24 h Post: 24 h, 48 h
	(4)	10 (n.r.)	19.00 ± 1.00	U-20 Brazilian first division soccer league	Two soccer matches with three days of recovery between each match	Blood/Plasma creatine kinase concentration	Pre: 24 h Post: 1st match: 24 h, 48 h Post: 2nd match: 24 h, 48 h
	(33)	11 (11,0)	29.26 ± 4.52	Elite team in brazilian soccer	Three consecutive games	Blood C-reactive protein concentration	Pre: 5 days Post: 24 h, 48 h, 72 h
Combat/Judo	(37)	23 (23,0)	20.1 ± 4.7	National team of college judo athletes, black belt	(a) The test started with 2 ukes (judoka threw) separated by 10 m and a tori (thrower judoka) between them; (b) the test begins at a speed 6 km/h where the tori must move alternately by the ukes and throw them by ippon-seoi-nage; (c) the stages were standardized in 2 min by 1-min interval; (d) each complete stage added to 0.5 km·h21 in the velocity; (e) the test finishes when the judoka cannot maintain the pace set by the current stage; and (f) accounts to total score using only the complete stages	Blood lactate concentration	Pre: 0 min Post: 5 min, 10 min, 15 min
	(7)	32 (25,7)	18.0 ± 3.5	Black-belt judokas from the Spanish junior national team	(a) Warm-up (20 min): 5-min running at low-intensity index <9 in 6–20 Borg scale, 5-min stretching, 5-min sprint interval training all-out (10″ with 10″ of interval), 4-min judo falling simulation (ukemis), and 1-min interval. (b) Technical training (30 min): 10-min technique application (uchi-komi) all-out = 8 × (20″ with 10″ of interval), 2-min interval and 8 × (20″ with 10″ of interval), 10-min throw technique (nage-komi), and 2 × 4-min handgrip dispute (2-min interval). (c) Combat training (Randori—40 min): standing combat (tachi-waza) 7 × 4-min with 1-min interval and 1 × 5-min (change the opponent every combat)	Blood: Count of white blood cells (leukocytes), subsets (neutrophils, lymphocytes, monocytes, eosinophils, basophils, and platelets), and haematocrit amount	Pre: 90 min Post: 0, 1 h, 24 h
(Half-) Marathon	(38)	17 (11,6)	41 ± 6	Recreational runners	Competition was the World Half Marathon Championship in Valencia, Spain	Blood/Plasma creatine kinase and glutamate oxaloacetate transaminase concentration Perception of fatigue and pain were measured using a 150 mm visual analogue scales (VAS)	Pre: 24 h, 48 h Post: 24 h, 48 h
	(39)	16 (9,7)	36 ± 7	Recreational marathon runners	A marathon in a hot environment (thermal stress index = 28.3 ± 3.3°C and humidity −81%)	Blood/Serum creatine kinase, and lactate dehydrogenase concentration	Pre: 45 min Post: 24 h
Other sports	(40)	10 (10,0)	40 ± 6	Recreational triathletes; triathlon experience was 7 ± 3 years	First day: Training cycling: 94 km at an intensity of 70% VO2max including 6k of uphill time trial all-out. 1,800 meters of swimming in the sea at an intensity of 60-70% VO2max. Core training session 45 min) Second day: Training cycling: 156 km + 2,977 elevation meter. 60% of the route at 60%–70% VO2max and 40% of the route at 71%–85% VO2max (uphills). Joint mobility session, no cardiovascular and strength effort (45 min)	Perception of fatigue and pain were measured using a 150-mm visual analogue scale (VAS)	2 days: Pre: 1.25 h and third day in the morning
	(35)	22 (22,0)	Endurance athletes (triathletes and long-distance runners): 24.5 ± 5.4 Sprint athletes: 25.0 ± 3.4	Polish national teams	Treadmill - 3 min of stand followed by - walking for the first 3 min - a speed of 4 km/h, then increased to 8 km/h. Then, the treadmill speed increased by 2 km/h every 3 min until volitional exhaustion of the subject	Blood lactate, ammonia concentration	Pre: Rest Post: 0, 5, 10, 15, 20, and 30 min

**Table 2:** Characterization of infrared thermography assessment.

Sport	Article	Thermographic parameters	Body region of thermographic assessment	Time point of thermographic assessment	Camera	Analysis Method of thermographic parameters	Statistical method
Studies investigating response to a (partial) season; Soccer	(9)	Mean skin temperature	14 anterior ROIs and 14 posterior ROIs of the leg	Post: 48 h	T450 model thermal camera (FLIR1 Systems, Danderyd, Sweden) was used, with a precision of up to 0.05°C and emissivity of 0.98	Temperature asymmetries were considered when the athlete presented a difference of 1°C between the limbs and reported pain in one body region mean skin temperature asymmetries between corresponding ROIs in the contralateral limbs	Pearson correlation
	(36)	Skin Temperature	Anterior and posterior regions of the lower limbs	Pre: Season Post: Season after an interval of 72 h of inactivity (no training)	T420, FLIR Systems (Stockholm) with measuring range from −20 to +120°C, precision of 1%, sensitivity ≤0.05°C, an infrared spectral band of 7.5 to 13 μm, refresh rate of 60 Hz, autofocus, and resolution of 320 × 240 pixels	Hot zone Tsk percentage (≥33°C) symmetry angle equation were used to identify Tsk asymmetry	Spearman correlation and regression (no details reported)
	(12)	Average, minimum and maximal skin temperature	Anterior and posterior regions of the lower limbs (44 ROIs)	Post: 72 h	FLIR T435bx (FLIR Systems, Sweden) with a resolution of 320 × 240 pixels and thermal sensitivity ≤0.04°C/<40 mK, was placed 3 m away from the participants and at a perpendicular angle to them, around 60 cm height	Mean Tsk and mean Tsk asymmetries between corresponding ROIs in the contralateral limbs. For asymmetries, every bilateral ROI of the players were classified in a subgroup: low (<0.3°C) or high thermal asymmetry (≥0.3°C). Further calculation based on this subgroups	Product–moment correlations (Pearson *r*)
Studies investigating response to a (partial) season; Other sports, Sprinting, Taper period	(34)	Mean and standard deviation of skin temperature	Anterior and posterior regions of the lower limbs	08:00 and 09:00 and between 20:00 and 21:00 local time; approximately Pre: 1 h, Post: not derivable	FLIR SC640 IR camera (FLIR Systems Inc., model SC640, Sweden)with noise-equivalent temperature difference	Daily skin temperature changes	Spearman correlation
Soccer; Studies investigating response to ≤3 soccer games	(3)	Mean skin temperature	Anterior and posterior regions of the legs	Pre: 24 h Post: 24 h, 48 h	FLIR T420, FLIR Systems Inc., Wilsonville, OR, USA with a measurement range from −20°C to + 120°C, 2% accuracy, sensitivity ≤0.05°C, IR spectral band of 7.5 to 13 μ, refresh rate of 60 Hz, auto-focus and a resolution of 320 × 240 pixels	No further analysis	No further analysis
	(4)	Mean skin temperature	Anterior and posterior side of: right thigh, left thigh, right leg, left leg	Pre: 24 h Post 1st match: 24 h, 48 h Post 2nd match: 24 h, 48 h	FLIR, T420, Flir Systems Inc., Wilsonville, Oregon, USA), with a measurement range from −20 to 120°C, 2% accuracy, sensitivity <0.05°C, IR spectral band of 7.5–13 lm, refresh rate of 60 Hz, auto-focus and a resolution of 320 × 240 pixels	Delta Pre-Post of mean skin temperature	Spearman correlation
	(33)	Skin temperature	Lower limbs (detailed areas n.r.)	Post: 24 h, 48 h, 72 h	T1020, FLIR, Stockholm, with measuring range from − 20 to +120°C, sensitivity ≤0.02°C, refresh rate of 60 Hz, autofocus and FULL HD resolution, 1.5 m distance	Absolute and relative delta in temperature from Pre - Post using the thermal pixelgraphy method, considering pixels compatible with temperatures ≥33°C	Spearman correlation
Combat/Judo	(7)	Body skin temperature	Body skin temperature were calculated using the equation by Ramanathan: BST = 0.3 × [chest] + 0.3 × [upper arm] + 0.2 × [thigh] + 0.2 × [calf] 4 regions of interest (ROIs) (chest, posterior upper arm, anterior thigh, and anterior calf) on the right side of the body	Pre: 0 min, Post: 0 min, 90 min, 24 h	FLIR T335 thermal camera (FLIR Systems, Danderyd, Sweden) was used with a measurement range from 220 to +1208°C, 2% accuracy, sensitivity # 0.058°C, IR spectral band of 7.5–13 mm, refresh rate of 60 Hz, autofocus, and a resolution of 320 × 240 pixels	Delta Pre-Post of skin temperature	Logistic regression analysis and Pearson correlation
	(37)	Mean skin temperature	Mean skin temperature of the entire body was calculated using the 10 sites, according to the equation of Houdas and Ring (0.06 × forehead) + (0.12 × check) + (0.12 × abdomen) + (0.12 × subscapular) + (0.08 × posterior upper arm) + (0.06 × posterior forearm) + (0.125 × anterior-medial-thigh) + (0.075 × anterior calf) +(0.075 × posterior calf)	Pre: 0 min Post: 5 min, 10 min, 15 min	FLIR T440 (Flir Systems, Inc., Wilsonville, OR, USA) 320 × 240 pixels, thermal sensitivity, 50 mK, and accuracy of 62°C Low-intensity: 40 min at 65% HR_max_	Delta Pre-Post of mean skin temperature	Linear regression
(Half-)Marathon	(38)	Average temperature, the maximum temperature and the standard deviation	10 ROIs of the full body: (1) anterior upper limbs, (2) posterior upper limbs, (3) abdominal, (4) lumbar back, (5) anterior thigh, (6) posterior thigh, (7) knee, (8) popliteus, (9) anterior leg, and (10) posterior leg mean skin temperature was calculated using the modified equation of Newburg–Spealman (mean = 0.34 * abdomen + 0.15 * posterior forearm + 0.33*posterior thigh + 0.18*posterior leg)	Pre: 24 h, 48 h Post: 24 h, 48 h	E-60, 320 × 240 pixels, Flir Systems Inc. (Wilsonville, OR, USA) with noise-equivalent temperature difference (NETD)	Delta Pre-Post of mean skin temperature and average skin temperature of ROIs	Elastic-net penalized logistic regression model
	(39)	Mean skin temperature	13 ROIs of anterior and posterior limbs	Pre: 15 days, 45 min Post: 24 h and 6 days	T440, FLIR Systems, Wilsonville, OR, USA). The camera had a focal plane size of 320 × 240 pixels with a measurement uncertainty of ±2% and a thermal sensitivity of 0.04°C	Delta Pre (15 days) – Post (24 h) mean skin temperature	Multiple linear regression
Other sports	(40)	Mean temperature, maximum temperature and the standard deviation	8 ROIs from the upper and lower limbs	2 days: Pre: 1.,25 h and third day in the morning	E60, Flir Systems Inc. (Wilsonville, OR, USA) with noise-equivalent temperature difference (NETD) <0.05°C, focal plane sensor array size of 320 × 240, and measurement uncertainty of ± 2% of the overall operational temperature range	Delta Pre-Post of mean skin temperature and average skin temperature of ROIs	Multiple linear regression
	(35)	Mean skin temperature and standard deviation	Anterior and posterior lower limbs	Pre: Rest Post: 0, 5, 10, 15, 20, and 30 min	FLIR SC640 IR camera (FLIR Systems Inc., SC640 model, Sweden) 640 × 480 pixels, noise-equivalent temperature difference (NETD) <30 mK, temperature accuracy of ±2%	No further analysis	Pearson correlation

n.r., not reported; ROI, region of interest; Tsk, skin temperature.

Due to the variability in intervention periods and thereby methodological approaches (resulting in e.g., different statistics), studies were categorized based on the intervention time: (i) studies which conducted a single exercise, training session or investigating response to ≤ 3 soccer games (*n* = 9) (3, 4, 7, 33, 35, 37–40) and (ii) studies which encompassed frequent assessment of parameters across (at least parts of) a competitive season (*n* = 4) (9, 12, 34, 36).

### 3.2 Methodological approaches of infrared thermography

Regions of interest (ROIs) for IRT assessment included lower limbs only (*n* = 9) (3, 4, 9, 12, 33–36, 39), whole body (*n* = 3) (7, 37, 38), upper and lower limbs (*n* = 1) (40). The most assessed IRT related parameter was the mean skin temperature of different regions of interest (ROIs) [*n* = 7 (4, 7, 33, 37–40);]. Seven out of 13 studies analyzed differences in IRT related parameter between pre and post exercise and three studies investigated temperature asymmetries of contralateral ROIs (9, 12, 36). One study investigated daily IRT related parameter changes (34), while two studies conducted no further analysis (3, 35). Evaluation of IRT related parameters includes thermal pixelgraphy method (*n* = 1) (33), hot zone IRT related parameter percentage method (*n* = 1) (36) or averaging temperatures of the whole body by employing established equations (*n* = 3) (7, 37, 38). Additionally, one study investigated the relationship between load parameters and the post-training skin thermal patterns of the athletes (7). Carvalho et al. (9) used a cut-off value of 1°C difference in Tsk between contralateral limbs and the report of pain in one body region for further analysis. Majano et al. (12) grouped participants depending on thermal asymmetry in contralateral ROI of players with thresholds set at < 0.3°C for low asymmetry and ≥ 0.3°C for high asymmetry. Rodriguez Junior et al. (36) grouped participants according to their total distance traveled in half.

### 3.3 Response of IRT to (non-)sport specific exercise and relationship with internal load

**Figure 2:**
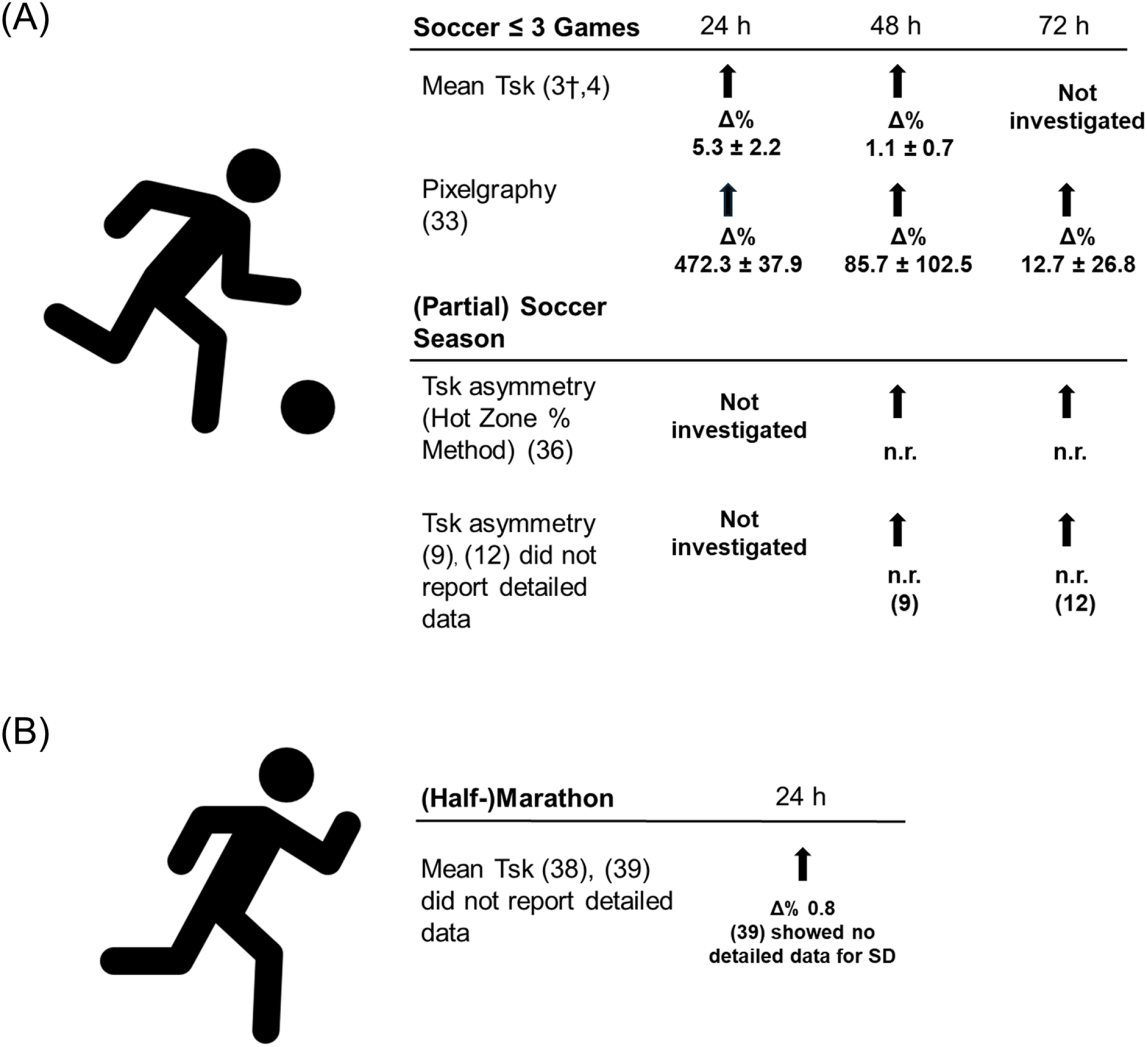
Changes in skin temperature in soccer and (half-)marathon. **(A)** Soccer ≤3 games and (partial) soccer season. **(B)** (Half-)Marathon. *Δ*% percentage changes based on mean values prior and after training or competition (self-calculated). _†_Single case study. Abbreviations: n.r., not reported in the study; Tsk, Skin temperature.

**Figure 3:**
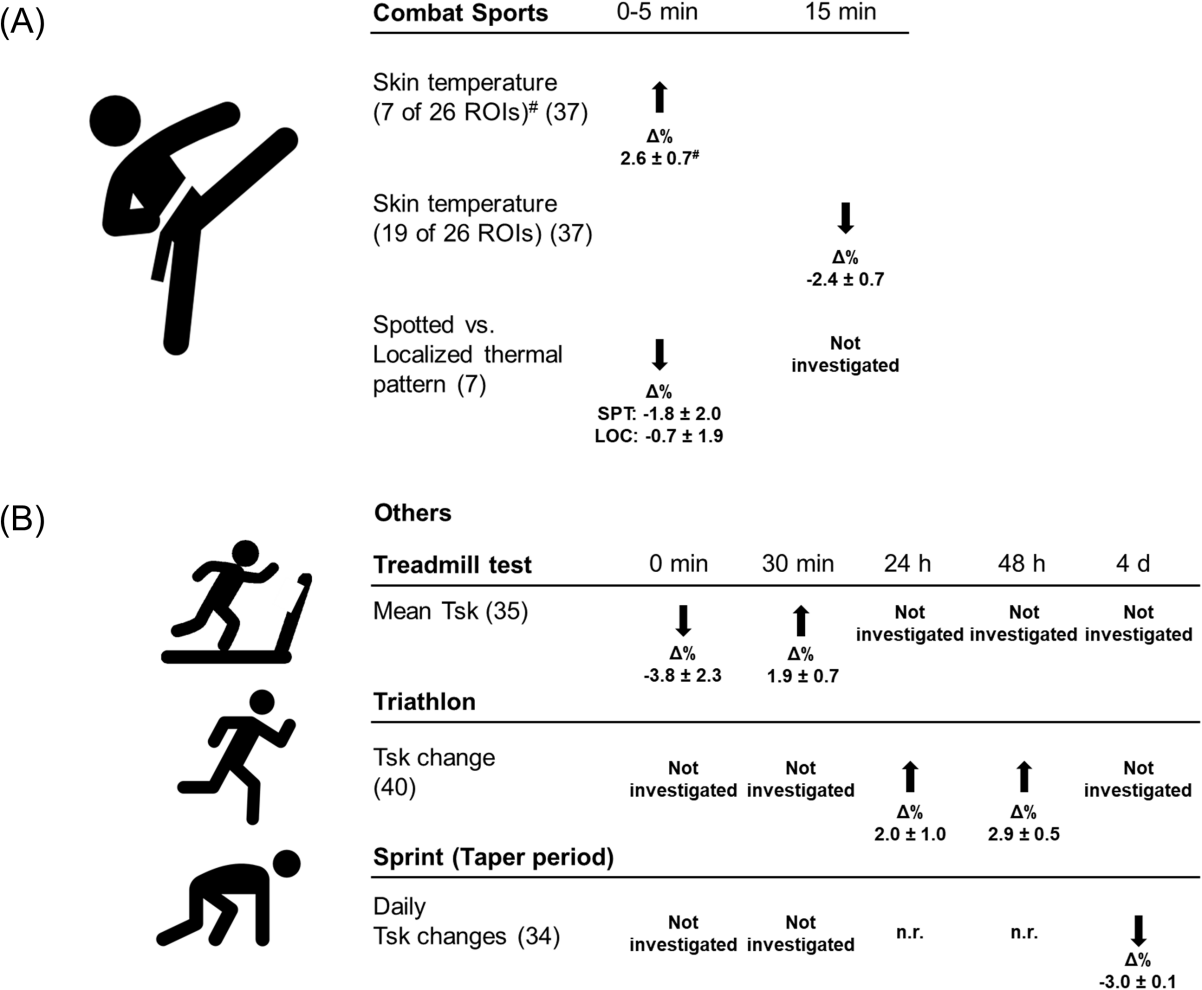
Changes in skin temperature in combat sports and other sports. **(A)** Combat sport. **(B)** Other sports (treadmill test, triathlon, sprint). *Δ*% percentage changes based on mean values prior and after training or competition (self-calculated). ^#^calculation based on 6 ROIs, as dataset only includes 6 ROIs with a Tsk increase. Abbreviations: n.r., not reported in the study; Tsk, Skin temperature.

Figures 2 and 3 summarize the response of IRT-related parameters to (non-)sport specific exercise.

Tables 3–5 represent the response of IRT related parameters following (non-)sport specific exercise and the relationship with internal load parameters.

**Table 3:** Infrared related parameter response and relationship with load parameter of studies in soccer investigating response to ≤3 soccer games and combat sport.

Sport	Article	Load				IRT related parameter				Relationship	
		Pre	Post	Δ% (self-calculated)	Sig.	Pre (in °C)	Post (in °C)	Δ% (self-calculated)	Sig.	Relationship	Sig.
Soccer	(3)	CK:	CK:		Single case: n.a.		24 h:		Single case: n.a.	Single case: n.a.	Single case: n.a.
		193 U/L	24 h: 1083 U/L	461.1		Thighs anterior: 31.5	Thighs anterior: 33.8	7.3			
			48 h: 414 U/L	114.5		Legs anterior: 31.2	Legs anterior: 33.3	6.7			
						Thighs posterior: 32.1	Thighs posterior: 34.6	7.8			
						Legs posterior: 31.0	Legs posterior: 33.2	7.1			
							48 h:				
							Thighs anterior: 31.8	1.0			
							Legs anterior: 31.5	1.0			
							Thighs posterior: 32.5	1.2			
							Legs posterior: 31.7	2.3			
	(4)	CK:	First match, 24 h:		All time points to pre: *P* = 0.000	Mean values	First match, 24 h, mean values (self-calculated):		Increase from Pre to Post match 1, 24 h all ROIs: *p* < 0.05 Increase from Pre to Post match 2, 24 h all ROIs: *p* < 0.05 Increase from Pre to Post match 2, 48 h anterior right and left thigh, anterior left leg: *p* < 0.05	CK:	
		221.8 ± 107.6 U/L	763.8 ± 294.5 U/L	244.4		(self-calculated):				Anterior:	Anterior:
			Second match, 24 h:			Anterior: 32.38 ± 3.50 Posterior: 32.50 ± 3.75	Anterior: 33.30 ± 0.38	2.86		Right thigh: *r* = 0.345	*p* < 0.01
			784.1 ± 298.8 U/L	253.5			Posterior: 33.33 ± 0.38	2.54		Right leg: *r* = 0.425	*p* < 0.01
										Left thigh: *r* = 0.353	*p* < 0.01
			First match, 48 h:				Second match, 24 h, mean values (self-calculated):			Left leg: *r* = 0.428	*p* < 0.01
			526.4 ± 289.7 U/L	137.3							
			Second match, 48 h:				Anterior: 33.80 ± 0.30	4.40		Posterior:	
			672.2 ± 285.0 U/L	203.1			Posterior: 33.70 ± 0.30	3.69		Right thigh: *r* = 0.276	*p* < 0.05
										Right leg: *r* = 0.289	*p* < 0.05
							First match, 48 h, mean values (self-calculated):			Left thigh: *r* = 0.299	*p* < 0.05
										Left leg: *r* = 0.257	*p* < 0.05
							Anterior: 32.43 ± 0.45	0.15			
							Posterior: 32.63 ± 0.475	0.38			
							Second match, 48 h, mean values (self-calculated):				
							Anterior: 33.03 ± 0.35	2.01			
							Posterior: 32.78 ± 0.40	0.85			
	(33)	CRP (mg/L):	CRP (mg/L):			In pixels:	All in pixels:			CRP:	
			Game 1:				Game 1:			Game 1:	
		0.55 ± 0.17	24 h: 2.60 ± 0.86	372.73	*p* < 0.001	9.91 ± 1.36	24 h: 54.99 ± 8.55	454.89	*p* < 0.001	Post (24 h): *r* = 0.60	*p* ≤ 0.05
			48 h: 1.65 ± 0.51	200.00	*p* < 0.001		48 h: 10.84 ± 1.73	9.38	*p* < 0.04	Post (48 h): *r* = 0.69	*p* ≤ 0.05
			72 h: 0.56 ± 0.18	1.81	n.s. *p* > 0.10		72 h: 14.09 ± 2.11	42.18	*p* < 0.001	Post (72 h): *r* = 0.72	*p* ≤ 0.05
			Game 2:				Game 2:			Game 2:	
			24 h: 3.14 ± 0.51	470.90	*p* < 0.001		24 h: 61.02 ± 5.30	515.74	*p* < 0.001	Post (24 h): *r* = 0.43	n.s.
			48 h: 0.73 ± 0.22	32.73	*p* < 0.001		48 h: 14.41 ± 2.52	45.41	*p* < 0.04	Post (48 h): *r* = 0.29	n.s.
			72 h: 0.59 ± 0.22	7.27	n.s. *p* > 0.10		72 h: 10.51 ± 1.50	6.05	n.s. *p* > 0.07	Post (72 h): *r* = 0.66	*p* ≤ 0.05
			Game 3:				Game 3:			Game 3:	
			24 h: 3.02 ± 0.63	449.09	*p* < 0.001		24 h: 54.13 ± 9.79	446.22	*p* < 0.001	Post (24 h): *r* = 0.21	n.s.
			48 h: 1.96 ± 0.41	256.36	*p* < 0.001		48 h: 29.95 ± 4.87	202.22	*p* < 0.04	Post (48 h): *r* = 0.21	n.s.
			72 h: 0.71 ± 0.12	29.09	n.s. *p* > 0.10		72 h: 8.91 ± 2.04	−10.09	n.s. *p* > 0.07	Post (72 h): *r* = 0.18	n.s.
	Sum	CK: 2	2 out of 2: Increase (Single case) and significant increase (Both 24, 48 h)			3 out of 3 increases (2 sig., 1 single case) (24, 48, 72 h)					1 out of 1: Relationship with CK, CRP
		CRP: 1	1 out of 1 (24, 48 h)								
Combat	(7)	Leukocytes:	Leukocytes:			Spots Group:			Pre to Post 0 h:	Variation of	*p* = 0.34,
		6.3 ± 1.3	0 h: 8.5 ± 2.0	34.9	*p* ≤ 0.001	33.85 ± 0.56	0 h: Spots: 33.22 ± 0.52 Localized: 33.80 ± 0.86	0 h: Spots: −1.86 Localized: −0.69	Spots: *p* ≤ 0.001	leukocytes including	n.s.
			1 h: 10.2 ± 3.3	61.9	*p* ≤ 0.001	Localized Group:	1 h: Spots: 34.10 ± 0.56 Localized: 34.22 ± 0.74 24 h: Spots: 33.61 ± 0.40 Localized: 33.82 ± 0.65	1 h: Spots: 0.74 Localized: 0.55 24 h: Spots: −0.71 Localized: −0.62	Localized:	all data:	
			24 h: 6.5 ± 1.7	3.2	*p* ≤ 0.001	34.03 ± 0.69			*p* = 0.043	*R* = 0.16	
									Post 0 h: Spots lower body skin temperature than Localized: *p* = 0.016	Group results: Spots lower leukocytes and neutrophils than localized	*p* ≤ 0.001
	(37)	Lactate:	Lactate:			34.2 ± 0.5	5 min: 34.3 ± 0.6	0.29	Pre to 15 min:	Difference Tsk 5 min	*p* = 0.001
		1.1 ± 0.3 mmol/L	0 min: 6.4 ± 2.7	481.8	*p* ≤ 0.001		10 min: 33.8 ± 0.6	−1.17	*p* = 0.002, 5 min to 10 and 15 min: *p* < 0.001, *p* < 0.001	and Tsk baseline to lactate 0 min post: *r* = 0.66	
			5 min: 5.9 ± 2.4	436.4	*p* ≤ 0.001		15 min: 33.5 ± 0.6	−2.05			
			10 min: n.r.	n.r.	n.r.						
			15 min: 4.0 ± 1.9	263.6	*p* ≤ 0.001					Difference Tsk 10 min and Tsk baseline to lactate 0 min post: *r* = 0.55	*p* = 0.006
	Sum	Leukocytes: 1		1 out of 1: Significant increase		2 out of 2: Significant decreases (0 h, 15 min Post) 1 out of 1: Skin thermal pattern has impact on Tsk (0 h)				1 out of 1: Relationship with lactate 0 out of 1: Relationship with leukocyte count 1 out of 1: Skin thermal pattern has impact on leukocytes and neutrophils	
		Lactate: 1		1 out of 1: Significant increase (Post 0, 5, 15 min)							

CK, creatine kinase; CRP, C-reactive protein; n.a., not available; n.r., not reported; n.s., not significant; ROI, region of interest; Sig., significance; Tsk, skin temperature. Δ% percentage changes.

**Table 4:** Infrared related parameter response and relationship with load parameter for studies in (half-) marathon, triathlon and treadmill test.

Sport	Article	Load						IRT related parameter						Relationship	
		Pre		Post		Δ% (self-calculated)	Sig.	Pre (in °C)		Post (in °C)		Δ% (self-calculated)	Sig.	Relationship	Sig.
(Half-) Marathon	(38)^a^	48 h:	24 h:	24 h:	48 h:			48 h:	24 h:	24 h:	48 h:	48 h Pre to 24 h Post: Mean skin temperature: 0.77	Skin temperature Pre 48 h to Post 24 h: Increase in posterior upper limb: *p* < 0.001	Differences between measurement 24 h post half marathon and average of the measurements 24 h and 48 h before half marathon: posterior upper limb: ΔCK24 - ΔT24: *r* = 0.5	*p* = 0.04
		CK: 137.71 (U/L) ± 148.48	CK: 145.03 (U/L) ± 118.25	CK: 752.25 (U/L) ± 448.19	CK: 450.32 (U/L) ± 403.33	24 h Pre to 24 h Post: 418.69	Pre 24 h to Post 24 h: *p* < 0.001	Mean skin temperature (Newburg-Spielman):	Mean skin temperature (Newburg-Spielman):	Mean skin temperature (Newburg-Spielman):	Mean skin temperature (Newburg-Spielman):				
								32.26 ± 0.80	32.73 ± 0.76	32.51 ± 0.62	32.69 ± 0.68				
								Posterior upper limb:	Posterior upper limb:	Posterior upper limb:	Posterior upper limb:	Posterior upper limb:			
								31.11 ± 0.81	31.94 ± 0.96	31.89 ± 0.94	32.39 ± 0.81	2.51			
								Knee:	Knee:	Knee:	Knee:	Knee: 1.57			
								29.97 ± 1.29	30.97 ± 1.08	30.44 ± 0.97	30.74 ± 1.21				
		48 h: GOT (U/L): 33.67 ± 11.53	24 h: GOT (U/L): 34.52 ± 10.17	24 h: GOT (U/L): 74.56 ± 52.00	48 h: GOT (U/L): 64.3 ± 45.40	24 h Pre to 24 h Post: 115.99	24 h Pre to 24 h Post: *p* < 0.04							GOT: n.s.	
		48 h: Pain: 1.05 ± 1.26	24 h: Pain: 1.14 ± 0.93	24 h: Pain: 4.71 ± 3.18	48 h: Pain: 4.36 ± 3.01	24 h Pre to 24 h Post: 313.16	24 h Pre to 24 h Post: *p* < 0.001							knee: Δoverallpain 48 - ΔT48: *r* = 0.6	*p* < 0.01
		48 h: Fatigue: 1.51 ± 1.43	24 h: Fatigue: 1.89 ± 1.57	24 h: Fatigue: 6.54 ± 3.03	48 h: Fatigue: 5.46 ± 2.80	24 h Pre to 24 h Post: 246.03	24 h Pre to 24 h Post: *p* < 0.001							Fatigue: n.s.	
		48 h: CMJ height: 24.4 cm ± 4.1	24 h: CMJ height: 25.0 cm ± 4.9	24 h: CMJ height: 23.4 cm ± 4.0	48 h: CMJ height: 24.6 cm ± 4.5	24 h Pre to 24 h Post: −6.40	24 h Pre to 24 h Post: *p* < 0.01							CMJ: n.s.	
	(39)	CK:		CK:				n.r.		n.r.		n.a.	Significant increases from Pre 15 days to Post 24 h: All ROIs (except Semi-tendinous *p* < 0.01): *p* < 0.001		n.s. *p* > 0.21
		174.3 UI/L ± 136.4		1159.7 UI/L ± 699.7		565.35	*p* < 0.01								
		LDH:		LDH:											
		362.6 UI/L ± 99.9		438.0 UI/L ± 115.5		20.79	*p* = 0.02								
	Sum	CK: 2				2 out of 2: Significant increase (Post 24 h)		2 out of 2: Significant increase (Both post 24 h)						1 out of 1: Relationship with pain 1 out of 2: Relationship with CK 0 out of 1: Relationship with GOT, LDH, fatigue, CMJ height	
		GOT: 1				1 out of 1: Significant increase (Post 24 h)									
		LDH: 1				1 out of 1: Significant increase (Post 24 h)									
		Perception of pain: 1				1 out of 1: Significant increase (Post 24 h)									
		Perception of fatigue: 1				1 out of 1: Significant increase (Post 24 h)									
		CMJ height: 1				1 out of 1: Significant decrease (Post 24 h)									
Triathlon	(40)^a^			24 h:	48 h:	24 h Post:		Knee: 28.4 ± 1.1 Posterior leg: 29.2 ± 0.8		24 h: Knee: 29.3 ± 0.5		3.17	*p* < 0.01	Δ48 h knee fatigue and Δ48 h anterior knee Tsk: *R*^2^ = 0.5 (inverse) No relationship with pain and overall fatigue	*p* = 0.03
		Fatigue; Overall: 2.2 ± 2.2		Fatigue; Overall: 4.5 ± 3.1	Fatigue; Overall: 5.7 ± 3.2	Fatigue; Overall: 104.55	Overall: 24 h: *p* < 0.05, 48 h: *p* < 0.01			Posterior leg: 29.4 ± 0.8		0.68	n.s.		
										48 h: Knee: 29.4 ± 1.0		3.52	*p* < 0.01		
		Knee: 0.3 ± 0.5		Knee: 1.8 ± 2	Knee: 2.2 ± 2.4	Knee: 500.00	Knee: 24 h, 48 h: n.s.			Posterior leg: 29.8 ± 0.8		2.05	*p* < 0.05		
						48 h Post: Fatigue; Overall: 159.09 Knee: 633.33								Δ24 h posterior knee Tsk and reported cycling and running volume: R2 = 0.7	*p* < 0.01
		Pain: Overall: 2.2 ± 2.6		24 h: Pain; Overall: 3.5 ± 2.7	48 h: Pain; Overall: 3.9 ± 2.6	24 h Post: 59.09 48 h Post: 77.27	Pain; Overall: 24, 48 h: n.s.							Δ24 h posterior leg Tsk and reported cycling volume: R^2^= 0.44	*p* < 0.04
		Reported running, cycling and swimming volume: 28 ± 12 km, 159 ± 61 km and 5 ± 2 km					n.r.								
Treadmill Test	(35)	Lactate (mmol/L):		Lactate (mmol/L):			Pre to Post: n.r.	Endurance group:		Endurance group:			Pre to Post comparison n.r.	Skin temperature - Lactate correlations: Between the 10th and 30th minute of recovery; r ranging from −0.54 to −0.45	*p* < 0.05
		Endurance group:		Endurance group:				31.74 ± 0.73		0 min: 30.02 ± 1.07		−5.42			
		1.05 ± 0.35		0 min: 10.17 ± 1.49		868.57				5 min: 31.64 ± 1.08		−0.32			
				5 min: 8.96 ± 1.80		753.33				10 min: 31.78 ± 1.17		0.13			
				10 min: 7.43 ± 1.91		607.62				15 min: 31.93 ± 1.12		0.60			
				15 min: 6.15 ± 1.73		485.71				20 min: 32.12 ± 1.06		1.20			
				20 min: 5.04 ± 1.67		380.00				30 min: 32.49 ± 0.81		2.36			
				30 min: 3.58 ± 1.24		240.95									
		Ammonia (mmol/L):		Ammonia (mmol/L):			Pre to Post: n.r.	Sprint group: 32.26 ± 0.58		Sprint group:			Pre to Post comparison n.r.	Skin temperature - Lactate correlations: Between the 10th and 30th minute of recovery; r ranging from −0.54 to −0.45	*p* < 0.05
		Endurance group:		Endurance group:						0 min: 31.55 ± 0.75		−2.20			
		21.18 ± 6.29		0 min: 75.64 ± 11.51		257.13				5 min: 32.39 ± 0.61		0.40			
				5 min: 65.55 ± 10.30		209.49				10 min: 32.32 ± 0.58		0.19			
				10 min: 52.55 ± 8.72		148.11				15 min: 32.26 ± 0.66		0.00			
				15 min: 43.36 ± 7.68		104.72				20 min: 32.38 ± 0.58		0.37			
				20 min: 34.27 ± 6.50		61.80				30 min: 32.70 ± 0.53		1.36			
				30 min: 27.55 ± 7.43		30.08									
		Lactate (mmol/L):		Lactate (mmol/L):										n.s. correlations between Tsk and ammonia	n.s.
		Sprint group:		Sprint group:											
		1.18 ± 0.30		0 min: 10.14 ± 1.40		759.32									
				5 min: 9.03 ± 1.60		665.25									
				10 min: 7.55 ± 1.44		539.83									
				15 min: 6.10 ± 1.48		416.95									
				20 min: 4.95 ± 1.24		319.49									
				30 min: 3.50 ± 1.03		196.61									
		Ammonia (mmol/L):		Ammonia (mmol/L):											
		Sprint group:		Sprint group:											
		18.91 ± 3.48		0 min: 76.73 ± 9.03		305.76									
				5 min: 68.45 ± 10.64		261.98									
				10 min: 57.36 ± 9.73		203.33									
				15 min: 48.55 ± 8.64		156.74									
				20 min: 40.09 ± 6.67		112.00									
				30 min: 29.82 ± 5.31		57.69									
	Sum	Ammonia: 1			n.r.			1 out of 2: Significant increase (Post 24 h and Post 48 h) 1 out of 2: Increase						1 out of 1: Relationship with fatigue (knee), lactate, reported training volume 0 out of 1: Relationship with ammonia, pain, overall fatigue	
		Lactate: 1			n.r.										
		Perception of pain: 1			0 out of 1: Significant (Post 24, 48 h)										
		Perception of fatigue: 1			1 out of 1: Significant increase (Post 24, 48 h; Overall fatigue)										
		Reported training volume: 1			n.r.										

CMJ, countermovement jump; CK, creatine kinase; GOT, glutamate oxaloacetate transaminase; LDH, lactate dehydrogenase; n.a., not available; n.r., not reported; n.s., not significant; ROI, region of interest; Sig., significance; Tsk, skin temperature. Δ% Percentage changes.

^a^ Only significant results.

**Table 5:** Characteristics of (partial) season and relationship with internal load parameter in studies investigating response to a (partial) season.

Sport	Article	Characteristics of (partial) season	Thermographic region	Relationship with internal load			
Soccer	(9)	CK: 805.7 ± 560.5 IU/L, mean time in a match (min): 90.16 ± 10.74, total distance covered (m): 9017.04 ± 1621.48, high-intensity distance (m): 1140.90 ± 494.61, total number of high-intensity acceleration (m): 15.60 ± 7.68 and total number of high-intensity deceleration (m): 22.60 ± 8.97	Lower limb anterior total area	CK: *r* = 0.03	n.s.	Pain:	
						*r* = −0.18	n.s.
						Fatigue:	
						*r* = −0.19	n.s.
						Recovery:	
						*r* = 0.13	n.s.
			Lower limb posterior total area	CK: *r* = 0.10	n.s.	Pain:	
						*r* = −0.13	n.s.
						Fatigue:	
						*r* = −0.19	n.s.
						Recovery:	
						*r* = 0.10	n.s.
	(36)	Tsk: 72 h post season: *p* = 0.0001	Anterior region of lower limbs	Group 1:			
				Symmetry angle of peak force: *r* = 0.44			n.s.
				Symmetry angle of impulse: *r* = 0.31			n.s.
				Symmetry angle of the rate of force development: *r* = 0.62			*p* = 0.05, r^2^ = 0.39
			Posterior region of lower limbs	Group 1:			
				Symmetry angle of peak force: *r* = 0.13			n.s.
				Symmetry angle of impulse: *r* = 0.14			n.s.
				Symmetry angle of the rate of force development: *r* = 0.19			n.s.
			Anterior region of lower limbs	Group 2:			
				Symmetry angle of peak force: *r* = 0.61			n.s.
				Symmetry angle of impulse: *r* = 0.22			n.s.
				Symmetry angle of the rate of force development: *r* = −0.13			n.s.
			Posterior region of lower limbs	Group 2:			
				Symmetry angle of peak force: *r* = 0.22			n.s.
				Symmetry angle of impulse: *r* = 0.15			n.s.
				Symmetry angle of the rate of force development: *r* = 0.70			*p* = 0.03, *r*^2^ = 0.48
	(12)^a^	We refer to the article of Majano et al. (12).	Outer upper thigh	Asymmetry: Rest: *r* = −0.172			*p* ≤ 0.01
				Mean skin temperature: Muscle Soreness: *r* = −0.230			*p* ≤ 0.05
			Central upper thigh	Mean skin temperature: Muscle Soreness: *r* = −0.217			*p* ≤ 0.05
			Outer front thigh	Asymmetry: Muscle soreness: *r* = 0.234			*p* ≤ 0.05
			Central front thigh	Asymmetry: Rating of perceived exertion: *r* = −0.181			*p* ≤ 0.01
			Front adductor	Asymmetry: Muscle soreness: *r* = 0.174,			*p* ≤ 0.01
				Stress: *r* = 0.260			*p* ≤ 0.05
				Rest: *r* = −0.181			*p* ≤ 0.01
				Mean skin temperature: Stress: *r* = −0.161			*p* ≤ 0.01
			Inner front thigh	Mean skin temperature: Stress: *r* = −0.186			*p* ≤ 0.01
			Inner back leg	Mean skin temperature: Rest: *r* = −0.202			*p* ≤ 0.05
			Knee	Asymmetry: Rating of perceived exertion: *r* = 0.242 Mean skin temperature: Rest: *r* = −0.201, Rating of perceived exertion: *r* = 0.209			*p* ≤ 0.05
			Ankle	Mean skin temperature: Stress: *r* = 0.153			*p* ≤ 0.01
Sprinting	(34)	The baseline level of CK at the beginning of the camp was 146 ± 62 U/L in the female athletes and 236 ± 121 U/L in the male athletes. In both groups CK levels peaked on the second day (*p* < 0.01, female: Δ = 178, male: Δ = 163). The second increase in CK level in the male group occurred between 8th and 11th day (*p* < 0.01, 8th: Δ = 156, 9th: Δ = 209, 10th: Δ = 274, 11th: Δ = 188) with a peak value on the 10th day (510 ± 121 U/L, *p* < 0.01). In the case of the female group, there was no further increase in CK levels by the end of the camp.		CK: n.r.			n.s.
	Sum	CK: 2 Subjective load: 2 CMJ related parameters (RFD, impulse, peak force): 1		1 out of 2: Relationships (positive and negative) with various subjective load parameters (e.g., fatigue, pain, stress, sleep) 0 out of 2: Relationship with CK 1 out of 1: Relationships with RFD (same study showed no relationship with impulse and peak force)			

CMJ, countermovement jump; CK, creatine kinase; CRP, C-reactive protein; n.r., not reported; n.s., not significant; RFD, rate of force development; Tsk, skin temperature.

^a^ Only significant results.

### 3.4 Risk of bias

Table 6 summarizes the risk of bias according to QUADAS-2.

**Table 6:** Risk of bias assessment.

Study	Risk of bias				Applicability concerns		
	Patient selection	Index test	Reference standard	Flow and timing	Patient selection	Index test	Reference standard
(3)	HR	LR	LR	LR	LR	LR	LR
(4)	LR	LR	LR	LR	LR	LR	LR
(7)	UR	LR	LR	LR	UR	LR	LR
(9)	LR	LR	LR	LR	LR	LR	LR
(12)	LR	LR	LR	LR	LR	UR	LR
(33)	LR	LR	LR	LR	LR	LR	LR
(34)	LR	LR	LR	LR	LR	LR	LR
(35)	LR	LR	LR	LR	LR	LR	LR
(36)	UR	LR	LR	LR	LR	LR	LR
(37)	LR	LR	LR	LR	UR	LR	LR
(38)	LR	LR	LR	LR	LR	LR	LR
(39)	LR	LR	LR	LR	LR	UR	LR
(40)	LR	LR	LR	LR	LR	LR	UR

HR, high risk; LR, low risk; UR, unclear risk.

The first domain pertaining patient or participant selection indicates one study (3) with a high risk. De Andrade Fernandes (3) conducted a single-case study. Two studies were classified as unclear risk: Brito et al. (7) did not provide information of medical history, which could have been a part of the applied TISEM Checklist. Rodrigues Júnior (36) et al. utilized inclusion and exclusion criteria, but omitted information about application factors such as ointments or therapies. Applicability concerns regarding patient selection were categorized as unclear risk in studies by Brito et al. (7) and Gomes Moreira et al. (37), due to the potential inclusion of athletes under the age of 18, as indicated by the reported standard deviation of participants’ ages. The remaining 11 studies (3, 4, 7, 9, 12, 33–40) incorporate athletes who were suitable for the research question. In the second domain referring to the index test, all 13 studies (3, 4, 7, 9, 12, 33–40) showed a low risk of bias, while there are two studies with unknown risk of applicability concerns. The study of Rojas-Valverde (39) was classified as unclear for applicability, as they reported a cleaning and drying process before IRT assessment but did not provide information about the drying method. Majano et al. (12) classified body areas of every athlete depending on asymmetries on training days. Therefore, a one body part could be classified in low asymmetry group and another part could be classified in high asymmetry group. As there is no additional evidence supporting this methodological approach, it was classified as unclear.

Regarding the reference standard, all 13 studies (3, 4, 7, 9, 12, 33–40) applied parameters, which encompasses defined load states of the athletes and therefore showed no risk of bias. The investigation of Priego-Quesada (40) was classified as unclear risk of concerns in terms of applicability, as they analyzed reported volumes of running and cycling load without providing information how these volumes were recorded.

The fourth domain “Flow and Timing” reflected no risk of bias of any study, since there was no delay or follow-up periods between the index and reference tests.

## 4 Discussion

The aim of this article was to systematically review available literature on the response of IRT related parameters to (non-)sport specific exercise and relationship with internal load parameters. The main results are:

1. 11 out of 13 studies were published between 2019 and 2024, indicating rising interest in research around IRT related parameters following (non-)sport specific exercise in athletic populations.
2. Following (non-) sport-specific exercise in athletic populations, the majority of relevant studies showed a decrease in IRT related parameters within 15 min (*n* = 3) (7, 35, 37), while studies showed an increase in IRT related parameters following 30 min (*n* = 1) (35), 24 h (*n* = 5) (3, 4, 38–40), 48 h (*n* = 7) (3, 4, 9, 12, 33, 36, 40), and 72 h (*n* = 3) (9, 12, 33, 36) after exercise cessation.
3. Relationships between alterations in IRT related parameters and other internal load parameters are inconsistent across the literature. Synthetization of literature is impaired due to variety in used methodological approaches (e.g., calculated IRT related parameters, time points of measurement, assessed internal load parameters).

### 4.1 Soccer

#### 4.1.1 Studies investigating IRT related parameter response to ≤ 3 soccer games and relationship with other internal load parameters

Studies show a response in investigated IRT related parameters (i.e., change in mean Tsk; mean change after 24 h: 5.3%, after 48 h: 1.1%) (3, 4) and change in Tsk using thermal pixelgraphy method (mean change 24 h: 472.3%, after 48 h: 85.7%, after 72 h: 12.7%) (33) 24 h, 48 h and 72 h following a soccer match.

Studies investigating a relationship between mean Tsk and CK (24 and 48 h: anterior leg; mean r = 0.388, *p* ≤ 0.01; posterior leg; mean r = 0.280, *p* ≤ 0.05) (4) as well as Tsk evaluated through the thermal pixelgraphy method and CRP after one game (24 h post: r = 0.60, *p* ≤ 0.05; 48 h post: r = 0.69, *p* ≤ 0.05; 72 h post: r = 0.72, *p* ≤ 0.05) and after 2 consecutive games (72 h post: r = 0.66, *p* ≤ 0.05) (33).

Studies examined a relationship between mean Tsk and CK at a specific time point (24 and 48 h: anterior leg; mean r = 0.388, *p* ≤ 0.01; posterior leg; mean r = 0.280, *p* ≤ 0.05) (4) as well as Tsk evaluated through the thermal pixelgraphy method and CRP after one game (24 h post: r = 0.60, *p* ≤ 0.05; 48 h post: r = 0.69, *p* ≤ 0.05; 72 h post: r = 0.72, *p* ≤ 0.05) and with CRP after 2 consecutive games (72 h post: r = 0.66, *p* ≤ 0.05) (25).

While these studies show that soccer games evoke alterations in IRT related parameters (with 24 h post showing the highest alteration) which seem to have a relationship with CK and CRP, literature is still scarce and further research on response of IRT related parameters and relationship with internal load parameters is needed.

#### 4.1.2 Studies investigating infrared thermography related parameters response to a (partial) season and relationship with other internal load parameters

Three studies investigated IRT related parameters (9, 12, 36) and showed that Tsk asymmetries using the “hot zone percentage method” (72 h post season: *p* = 0.0001) (36) and mean Tsk asymmetries (mean Tsk asymmetry over entire season: 1.58 ± 0.84) (9, 12) are altered 72 h after a competitive season (36) and 48 h (9) after soccer matches. Majano et al. (12) (72 h after soccer matches) did not represent altered data for Tsk asymmetries.

While a study investigating ≤ 3 soccer games showed a positive relationship between IRT related parameters and CK (4), a finding which is further supported by a single case study (3), the study of de Carvalho (9) indicated no relationship between CK and IRT related parameter (48 h after soccer matches: mean r = 0.07, no *p*-values available) (9). We can only speculate on the reasons for this difference, but the study of de Carvalho (9) exclusively included thermographic images in the data analysis that exhibited Tsk asymmetries of ≥1.0°C, while e.g., Majano et al. (12) utilized a Tsk of 0.5°C to distinguish between athletes indicating a high or low Tsk response. While it was previously argued to use Tsk asymmetries of ≥1.0°C as abnormal side-to-side difference (5, 25, 41), due to advancements in IRT device accuracy, nowadays a lower temperature difference is often used (25).

Two studies (9, 12) assessed relationship between Tsk related parameters and subjective load parameters such as perception of pain, recovery, stress or rest. One (12) out of two showed a relationship between subjective parameters. Majano et al. (12) reported positive correlations between mean skin temperature and muscle soreness (central upper thigh, outer front thigh: r = 0.230, *p* ≤ 0.05, respectively), RPE (knee: r = 0.209, *p* ≤ 0.05) and stress (ankle: r = 0.153, *p* ≤ 0.01) as well as for thermal asymmetry between bilateral ROIs and muscle soreness (outer front thigh: r = 0.234, *p* ≤ 0.05; front adductor: r = 0.174, *p* ≤ 0.01) and RPE (knee: r = 0.242, *p* ≤ 0.05). They indicated negative correlations between mean skin temperature and rest (inner back leg: r = −0.202, *p* ≤ 0.05; knee: r = −0.201, *p* ≤ 0.05), as well as between thermal asymmetry in bilateral ROIs and rest quality (outer upper thigh: r = −0.172, *p* ≤ 0.01) and RPE (central front thigh: r = −0.181, *p* ≤ 0.01) (12). While we can only speculate on the reasons for this difference, Carvalho et al. (9) exclusively included athletes who reported pain scores above a threshold of 4, while the study of Majano did not use a threshold for pain scores (12). The difference in the used thresholds for pain scores in the studies (9, 12) could explain the different results.

A strong correlation between Tsk asymmetries and rate of force development assessed using a countermovement jump (mean r = 0.66, mean r^2^ = 0.44, mean *p* = 0.04) were examined following a competitive soccer season (36). The same study did not find relationships between the calculated impulse and peak force.

Collectively, but taken into consideration that literature is scarce, studies show that IRT related parameters are altered following a (partial) soccer season, which is in line with literature investigating IRT related parameters response to ≤3 soccer games. IRT related parameters seem to have a relationship with internal load such as subjective parameters and RFD measured by CMJs in studies investigating a (partial) soccer season. Interestingly, relationships between CK and IRT related parameters could not be established in studies investigating a (partial) soccer season, which could be due to different methodological approaches. However, as literature is scarce, more research is needed on the response of IRT related parameters and their relationship with internal load parameters.

### 4.2 (Half-)marathon

2 out of 2 studies showed elevations in IRT related parameters [Differences in mean skin temperature and ROIs (38, 39)], 24 h after a (half-)marathon in upper and/or lower leg muscles compared to time points prior to the competition (i.e., 48 h prior or 15 days prior) (38, 39).

Relationships between IRT related parameters and CK are non-conclusive following a (half-)marathon. While one study (38) showed a relationship between mean Tsk and CK (24 h post and average of 24 and 48 h post half-marathon, respectively; posterior upper limb: r = 0.5, *p* = 0.04) after a half-marathon, another could not identify a relationship between differences in mean Tsk and CK (*p* > 0.21) 24 h after exercise cessation (39). While we can only hypothesize on the reasons for the absence of a relationship in the study of Rojas-Valverde (39), Pérez-Guarner et al. (38) collected IRT related parameter data 24 and 48 h before a half-marathon, whereas Rojas-Valverde (39) analyzed IRT related parameter data from 15 days prior to a marathon.

Additionally, the hot environment in the study of Rojas-Valverde et al. (39) could confound Tsk results. There is evidence indicating that hot conditions may impair physiological responses (42), suggesting that Tsk could also be affected. Future research is required to understand how environmental conditions could impact Tsk responses.

Mean Tsk indicated a relationship with perception of overall pain (48 h post and average of 24 and 48 h post half-marathon: r = 0.6, *p* < 0.01) (38). Other analysis of the herein included articles did not show a relationship of mean Tsk with lactate dehydrogenase (39), glutamate oxaloacetate transaminase, fatigue, and CMJ height (38) following a (half-)marathon.

Collectively, there is evidence that IRT related parameters are altered 24 h after a (half-)marathon, yet synthetization of relationships with other internal load parameters is impaired due to low number of studies with different methodological approaches.

### 4.3 Combat sports

Two studies (7, 37) investigated IRT related parameters following combat sports and their relationship with other internal load parameters are difficult to summarize as they use different methodological approaches (e.g., measured IRT related parameters, internal load parameters, exercises).

Out of all studies in this review, the study of Brito et al. (7) reported different skin thermal patterns denoted as “spots” (SPT) and “localized” (LOC). The study showed lower temperatures for SPT and LOC immediately post training (7).

The study of Gomes Moreira et al. shows that skin temperature changes differ with respect to the region of interest in the minutes after cessation of exercise (37). While 7 regions of interest show an increase in skin temperature following 5 min of exercise (without an increase of mean skin temperature), 15 min post exercise 19 out of 26 regions of interest show a significant decrease in temperature (37). Gomes Moreira et al. thereby argues that post-training epithelial temperature is sensitive to organic variations and proposes that IRT related parameters can be applied as an indication of the intensity exerted after exertion (37), yet more studies are required to strengthen this assumption.

One of the main results of Brito et al. (7) is that skin thermal pattern, and not body skin temperature, defined by the equation of Ramanathan (43), correlates with internal load parameters. Specifically, Brito et al. (7) showed that a spotted skin pattern has a lower skin temperature (*p* = 0.016), blood leukocytes and neutrophils concentration (*p* ≤ 0.001) post-training compared to a localized skin thermal pattern. While skin thermal patterns have been investigated in other studies with athletic populations e.g., during exercise (44, 45), Brito et al. (7) acknowledges that more studies are needed which investigate the relationship between skin thermal pattern and immune response following exercise.

The main result of the study of Gomes Moreira et al. (37) shows that concentration of blood lactate at the end of a judo specific incremental test can be explained by the mean Tsk variation 5 (r = 0.66, *p* = 0.001) and 10 min (r = 0.55, *p* = 0.001) after the test. While literature is scarce, further investigations in athletic populations are needed, IRT related parameters could be useful to determine lactate post exercise non-invasively.

### 4.4 Other sports

Following a non-specific treadmill test, sprint and endurance athletes showed lower mean Tsk immediately after, (Tsk change in%; endurance group: −5.42 at 0 min, −0.32 at 5 min; sprinter group: −2.20 at 0 min) and a rise in mean Tsk 30 min post exercise (Tsk change in%; endurance group: 2.36 at 30 min, sprinter group: 1.36 at 30 min) (35). After treadmill testing, IRT related parameters indicated a relationship with lactate between the 10th and 30th minute after the testing (r ranging from −0.54 to −0.45, *p* < 0.05), but no relationship with ammonia (35).

In a training camp, triathletes showed an increase in all ROIs (Tsk change in%, e.g., knee: after 24 h: 3.17, after 48 h: 3.52; posterior leg: after 24 h: 0.68, 48 h: 2.05). IRT related parameters indicated an inverse relationship with the perception of knee fatigue (differences in knee fatigue and anterior knee Tsk: R2 = 0.5, *p* = 0.03) after two days of training, while no relationships were indicated with perception of pain and overall fatigue (40).

During a 10 day training camp in sprinters which included not further specified “very high intensity and low volume training”, a study noted a significant, continuous decrease in IRT related parameters (skin temperature) measured in the morning and evening for both genders (baseline: males 33.7 ± 0.4°C, females 32.8 ± 0.6°C; day 4: males 32.7 ± 0.3°C, females 31.8 ± 0.6°C, *p* < 0.05) (34). This downward trend persisted in males from day 6 till the end of the study and females from day 8 to the 10th day (*p* < 0.01) (34). While the authors (34) indicated an increase in CK concentration in male and female athletes on day two (*p* < 0.01) and a second CK concentration increase in male athletes between day 8 and 11 (*p* < 0.01), they did not exhibit a correlation between IRT related parameter and CK.

### 4.5 Limitations

This systematic review highlighted concerns about the lack of well-designed, and appropriately reported research in this field as indicated by our risk of bias assessment, as several studies neglected to provide transparent and complete information on methodological standardization. In accordance with Fernández-Cuevas et al. (25) and Moreira et al. (23), appropriate measurements, adherence to operational standards and consistent reporting are essential to ensure the interpretability of data across studies. As systematic reviews rely on the available research, this inconsistency in reporting represents a notable limitation. An additional limitation is the exclusion of studies involving participants under the age of 18, which may have led to the omission of insightful research. This exclusion criterion was established before the search process due to the potential influence of puberty on skin temperature (26). Accordingly, further research is required to investigate post-exercise skin temperature variations across different age groups, as suggested by Fernández-Cuevas et al. (25). Finally, our results only allow limited conclusions regarding the applicability of IRT to assess internal load in sports practice due to an inconsistency in employed methodological approaches (e.g., in assessed IRT parameters, blood-based parameters) and more research is needed which elucidated physiological mechanisms resulting in an alteration in IRT related parameters.

### 4.6 Recommendations for future research

The present review reveals that more studies are being performed on the use of IRT to assess internal load in different athletic populations since 2019 and that different IRT related parameters are altered following training and/or competition in athletic populations. Physiological mechanisms explaining IRT alterations in athletic populations following training and/or competition are not fully elucidated and need further investigations. From a physiological perspective, micro-damage to muscle cells leads to the release of damage-associated molecular patterns that stimulates resident cells to produce pro-inflammatory mediators (14). These mediators induce vasodilation and alter vessel permeability, increasing blood flow and promote edema (14). Additionally, stimulated endothelial cells express cell adhesion molecules and produce chemoattractant mediators, leading to the infiltration of leukocytes (14, 17, 46). The accumulation of leukocytes is a significant source of cytokines such as TNF-α, interleukin-1β and interleukin-6 (17). Research suggest that these cytokines exhibit pyrogenic properties (47, 48). In consequence, the primary inflammatory responses stimulate phagocytosis and the activation of the complement system to augment tissue repair (14, 16, 17, 46, 49, 50). These pathways may enhance local metabolic rates, substrate utilization, and energy generation, facilitating heat transfer from deeper tissue layers to the body surface, ultimately raising skin temperature (3, 6, 8, 33). Consequently, especially inflammatory parameters (such as CRP, pentraxin-3, prostaglandins, interleukins, TNF-α) (14–16, 49, 51, 52) need to be assessed in future studies.

As some studies in our review did not describe external load to which athletes were exposed in detail, comparisons between studies are impaired. To better compare future studies, we recommend researchers to report details on performed exercises which (depending on the sport) e.g., can include derivatives of GPS-data such as velocity, or accelerations. Additionally, future studies should control or at least report potential effect of factors known to confound IRT, such as food intake prior to image taking (25, 53), or body mass index (54). Our review reveals that different parameters can be calculated from IRT, including e.g., differences of mean Tsk, Tsk asymmetry, calculations of entire body skin temperature. While it is currently still unclear which IRT related parameters has the best relationships with other internal load parameters, it seems recommendable to assess and report a variety of IRT related parameters. To advance our understanding, we recommend also to report such relationships between IRT related parameters and other internal load variables if the relationship is non-existing.

## 5 Conclusions

Quantifying internal loads holds a key role to individualize training procedures, yet is often impaired e.g., due to invasiveness of methodological approaches, a limitation which might be overcome by IRT related parameters. Our systematic review reveals that the majority of relevant studies showed a decrease in IRT related parameters within 15 min, while increases in IRT related parameters are reported following 30 min, 24 h, 48 h, and 72 h after exercise cessation. Synthetization of the literature regarding relationship of IRT related parameters with other internal load parameters is impaired due to variety in used methodologies and is thereby non-conclusive across different sports. Future studies should carefully follow established recommendations to standardize IRT analyses and available literature. It seems recommendable to investigate the relationships with parameters known to elicit temperature in more detail. Athletes and coaches might detect changes in IRT related parameters following exercise cessation, but detailed physiological mechanisms leading to such change are currently unclear and it seems recommendable to use IRT parameters in conjunction with other load parameters.

## Data Availability

The original contributions presented in the study are included in the article/Supplementary Material, further inquiries can be directed to the corresponding author.
