# Supplementary material for: Response of infrared thermography related parameters to (non-)sport specific exercise and relationship with internal load parameters in individual and team sport athletes—a systematic review
Source: Front Sports Act Living. 2024 Dec 13;6:1479608. doi: 10.3389/fspor.2024.1479608 (PMC11671248; doi:10.3389/fspor.2024.1479608)
Supplement: Supplementary file 2 [file Table2.docx]

**Supplementary Table S2.** **Research terms used in the data bases.**

| Descriptors for infrared thermography | AND | Descriptors for sports | AND | Descriptors for load |
| --- | --- | --- | --- | --- |
| thermography |  | athlete |  | workload* |
| OR |  | OR |  | OR |
| infrared thermography |  | athletes |  | load* |
| OR |  | OR |  | OR |
| infrared thermal imaging |  | sports |  | duration* |
| OR |  | OR |  | OR |
| thermal image* |  | sport |  | frequenc* |
| OR |  | OR |  | OR |
| thermal imaging |  | rower |  | stress |
| OR |  | OR |  | OR |
| thermometry |  | football |  | strain |
| OR |  | OR |  | OR |
| skin temperature |  | rugby |  | train* |
| OR |  | OR |  | OR |
| thermographic changes |  | running |  | compet* |
| OR |  | OR |  | OR |
| infrared image* |  | runner |  | recovery |
| OR |  | OR |  | OR |
| infrared imaging |  | sailing |  | volume |
| OR |  | OR |  | OR |
| temperature mapping |  | shooting |  | intensit* |
| OR |  | OR |  | OR |
| temperature mappings |  | skating |  | stress |
| OR |  | OR |  | OR |
| thermo* |  | skater |  | congestion |
|  |  | OR |  | OR |
|  |  | snowboard |  | saturation |
|  |  | OR |  | OR |
|  |  | soccer |  | exposure* |
|  |  | OR |  | OR |
|  |  | swimming |  | jump* |
|  |  | OR |  | OR |
|  |  | swimmer |  | "rating of perceived exertion" |
|  |  | OR |  | OR |
|  |  | taekwondo |  | RPE |
|  |  | OR |  | OR |
|  |  | tennis |  | self-report* |
|  |  | OR |  | OR |
|  |  | trampoline |  | "self report" |
|  |  | OR |  | OR |
|  |  | triathlon |  | diary |
|  |  | OR |  | OR |
|  |  | triathlete |  | diaries |
|  |  | OR |  | OR |
|  |  | volleyball |  | questionnaire* |
|  |  | OR |  | OR |
|  |  | polo |  | survey* |
|  |  | OR |  | OR |
|  |  | weightlifting |  | scale* |
|  |  | OR |  | OR |
|  |  | weightlifter |  | journal |
|  |  | OR |  | OR |
|  |  | wrestling |  | journals |
|  |  | OR |  | OR |
|  |  | wrestler |  | inventory |
|  |  | OR |  | OR |
|  |  | athletics |  | inventories |
|  |  | OR |  | OR |
|  |  | "racquet sports" |  | self-evaluation |
|  |  | OR |  | OR |
|  |  | badminton |  | "self evaluation" |
|  |  | OR |  | OR |
|  |  | baseball |  | self-appraisal |
|  |  | OR |  | OR |
|  |  | basketball |  | "self appraisal" |
|  |  | OR |  | OR |
|  |  | biathlon |  | self-assessment |
|  |  | OR |  | OR |
|  |  | boxing |  | "self assessment" |
|  |  | OR |  | OR |
|  |  | boxer |  | self-rating |
|  |  | OR |  | OR |
|  |  | canoeing |  | "self rating" |
|  |  | OR |  | OR |
|  |  | cricket |  | subjective |
|  |  | OR |  | OR |
|  |  | ski |  | perceive* |
|  |  | OR |  | OR |
|  |  | skier |  | perceptual |
|  |  | OR |  | OR |
|  |  | curling |  | "profile of mood states" |
|  |  | OR |  | OR |
|  |  | cyclist |  | "daily analysis of life demands for athletes" |
|  |  | OR |  | OR |
|  |  | diving |  | wellbeing |
|  |  | OR |  | OR |
|  |  | diver |  | well-being |
|  |  | OR |  | OR |
|  |  | fencing |  | "well being" |
|  |  | OR |  | OR |
|  |  | fencer |  | wellness |
|  |  | OR |  | OR |
|  |  | hockey |  | health |
|  |  | OR |  | OR |
|  |  | (martial AND arts) |  | "physical state" |
|  |  | OR |  | OR |
|  |  | judo |  | "physical functioning" |
|  |  | OR |  | OR |
|  |  | kayak |  | "perceived recovery" |
|  |  | OR |  | OR |
|  |  | kayaking |  | "perceived strength" |
|  |  | OR |  | OR |
|  |  | karate |  | soreness |
|  |  | OR |  | OR |
|  |  | "nordic combined" |  | readiness |
|  |  | OR |  | OR |
|  |  | orienteer |  | vitality |
|  |  | OR |  | OR |
|  |  | orienteering |  | vigor |
|  |  | OR |  | OR |
|  |  | pentathlon |  | vigour |
|  |  | OR |  | OR |
|  |  | pentathlete |  | fatigue |
|  |  | OR |  | OR |
|  |  | rowing |  | overtrain* |
|  |  | OR |  | OR |
|  |  | biking |  | overreach* |
|  |  | OR |  | OR |
|  |  | hiking |  | heart rate variability |
|  |  | OR |  | OR |
|  |  | hiker |  | heart rate* |
|  |  | OR |  | OR |
|  |  | biker |  | TRIMP |
|  |  | OR |  | OR |
|  |  | cheerleader |  | lactate |
|  |  | OR |  | OR |
|  |  | cheerleading |  | urea |
|  |  | OR |  | OR |
|  |  | gymnastics |  | myoglobin |
|  |  | OR |  | OR |
|  |  | gymnast |  | uric acid |
|  |  | OR |  | OR |
|  |  | jogger |  | creatinine |
|  |  | OR |  | OR |
|  |  | jogging |  | testosterone |
|  |  | OR |  | OR |
|  |  | archery |  | tissue oxygenation |
|  |  | OR |  | OR |
|  |  | archer |  | pH |
|  |  | OR |  | OR |
|  |  | bobsleighing |  | creatine kinase |
|  |  | OR |  | OR |
|  |  | bobsledding |  | lactate dehydrogenase |
|  |  | OR |  | OR |
|  |  | equestrian |  | aspartate aminotransferase |
|  |  | OR |  | OR |
|  |  | golf |  | neuromuscular |
|  |  | OR |  | OR |
|  |  | golfer |  | asymmetr* |
|  |  | OR |  | OR |
|  |  | handball |  | muscular |
|  |  | OR |  | OR |
|  |  | luge |  | muscle* |
|  |  | OR |  | OR |
|  |  | "track and field" |  | electromyography |
|  |  |  |  | OR |
|  |  |  |  | EMG |
|  |  |  |  | OR |
|  |  |  |  | fatigue |
|  |  |  |  | OR |
|  |  |  |  | micro damag* |
|  |  |  |  | OR |
|  |  |  |  | dominance* |
|  |  |  |  | OR |
|  |  |  |  | injur* |
|  |  |  |  | OR |
|  |  |  |  | overuse |
|  |  |  |  | OR |
|  |  |  |  | soreness |
|  |  |  |  | OR |
|  |  |  |  | pain |
|  |  |  |  | OR |
|  |  |  |  | strain* |
|  |  |  |  | OR |
|  |  |  |  | sprain* |
|  |  |  |  | OR |
|  |  |  |  | musculoskeletal* |
